# Centenarians: Hip fractures and peripheral lower limb nerve blocks

**DOI:** 10.4103/0019-5049.65360

**Published:** 2010

**Authors:** Anand Rampure V Rao, Aneesh Lakshmanan, Anita Ajith, Sudheendra M Rao

**Affiliations:** Department of Anaesthesiology, Kannur Medical College, Anjarakandy, Kerala, India; 1 Department of Orthopaedics, Kannur Medical College, Anjarakandy, Kerala, India

Sir,

Elderly patients are most vulnerable to fractures due to osteoporosis.[1] Often anaesthesia for these set of patients is associated with higher risk.[2] We report a case of a 99-year-old fragile lady, who was to undergo hemi-arthroplasty for fractured neck of the left femur. Her poor cardiovascular status with severe left ventricular dysfunction and kyphoscoliosis of thoraco-lumbar spine made for a challenging anaesthetic management.

The patient was bedridden for last 2 days and must have been weighing around 45 kg, was having basal crepitations bilaterally on auscultation of the chest and fragile osteoporotic bones. Her other investigations were within normal limits except for serum creatinine (1.6 mg/dl), which was slightly above normal. Previously, she had come to us 6 months back, with a supracondylar fracture of the left humerus and had undergone fracture reduction and plating under a left brachial plexus block by the supra-clavicular approach using a peripheral nerve stimulator.

Informed consent was taken from the patient as well as her close relatives and the higher risk was explained to them with regard to anaesthesia. Sedative premedication was avoided and once the patient was in the theatre, an injection of pethidine 25 mg, slow intravenous, was given while supplementing O_2_ by a face mask. Patient was monitored with ECG (lead II and V5), SpO_2_, NIBP and CVP (right IJV). A lumbar epidural anaesthesia was initially planned, even though we were anticipating a technical difficulty in placing the epidural catheter, considering her difficult spinal anatomy. Two unsuccessful attempts were made with patient in the right lateral position, and that procedure was abandoned. Our next anaesthetic plan was the left lumbar plexus block.

A lumbar plexus block by the posterior approach was established with 15 ml of 0.5% bupivacaine and 10 ml of 2% plain lignocaine using a 10-cm insulated stimuplex needle and a peripheral nerve stimulator.[3] The onset of the block was around 15 min, after which the patient was placed in the right lateral decubitus position and the procedure was started. The whole surgical procedure was completed in an hour. The patient was on 35% O_2_ and 65% N_2_O by the face mask, spontaneously breathing, and additional bolus dose of pethidine 25 mg, iv, was supplemented during the procedure. The patient was haemodynamically stable throughout the procedure. Postoperative analgesia was maintained with NSAIDs.

With time, the number of geriatric patients who have to undergo surgical procedures which most often need the services of an anaesthesiologist is on the increase. They would have multiple co-morbidities associated with their age, which only get more complex with the increasing age. Patients undergoing hip fracture surgery constitute a high-risk group with considerable mortality and morbidity and an often protracted postoperative hospital stay. These patients often have a depleted intravascular volume in the perioperative period.[4] Also, in view of the poor cardiovascular status of our patient, we went for CVP monitoring, so that prompt treatment of any haemodynamic aberration could be instituted.

In certain subset of elderly patients, central neuroaxial blockade may not be the best anaesthetic choice, for example, in patients who would have undergone angioplasty with stenting and were on an antiplatelet regimen and general anaesthesia may not be that safe. We selected a single shot technique for establishing the lumbar plexus block, expecting the time duration of the block to be adequate for the procedure. However, a continuous lumbar plexus block using Tuohy-style tip needle with a catheter is an advanced regional technique, especially useful for postoperative pain management, for which adequate experience with the single shot technique is a prerequisite to ensure its efficacy and safety.

With increasing life expectancy, the anaesthesiologist comes across these set of patients more often, which not only tests the knowledge and experience but also the skill level. Present day anaesthesiologists should be familiar with the wide range of techniques in order to deal with such challenges so that anaesthesia could be made as safe as possible, especially in such a vulnerable age group.
